# Real-world localization of cancer in lungs with a commercially available folate receptor-targeted fluorescent agent for intraoperative molecular imaging

**DOI:** 10.1016/j.xjtc.2024.12.011

**Published:** 2025-01-23

**Authors:** Nicholas Baker, Evan T. Alicuben, Inderpal S. Sarkaria, Navid Ajabshir, Ryan M. Levy

**Affiliations:** a Department of Cardiothoracic Surgery, University of Pittsburgh School of Medicine and University of Pittsburgh Medical Center, Pittsburgh, Pa; b Department of Cardiovascular and Thoracic Surgery, UT Southwestern Medical Center, Dallas, Tex

**Keywords:** intraoperative molecular imaging, lesion localization, lung cancer, resection

## Abstract

**Background:**

Intraoperative molecular imaging (IMI) can improve lung nodule localization and the ability to perform sublobar resection. Following Food and Drug Administration approval of pafolacianine, we report on the integration of this folate receptor (FR)-targeted fluorescent agent into a minimally invasive thoracic surgery practice.

**Methods:**

Cases from June 2023 through January 2024 were reviewed. Patients with primary or metastatic cancer in the lung with plans for sublobar pulmonary resection were included. Preoperative computed tomography scans were used to determine lesion size and depth. Pafolacianine infusion was performed within 24 hours of surgery. The lung was inspected for fluorescence using the Stryker 1788 imaging system.

**Results:**

The study cohort comprised 39 patients (28 females and 11 males), with a median age of 68 years. The median lesion size was 13 mm (range, 5-32 mm), and median depth was 6.4 mm (range, 0-30 mm). Minimally invasive resection (robotic-assisted thoracoscopic surgery, n = 21; video-assisted thoracoscopic surgery; n = 18) was performed in all patients (segmentectomy, n = 15; wedge resection, n = 17; segmentectomy and wedge resection, n = 3; lobectomy, n = 4). In 11 patients, the primary lesion was not detectable under visual inspection with white light but was visualized with IMI. The final histology included primary lung cancer in 28 patients and metastatic cancer in 11 patients. All margins were negative.

**Conclusions:**

This report of early postmarketing experience with pafolacianine for cancer in the lung demonstrated a high rate of nodule localization. These early experiences further reinforce IMI as an adjunct to surgical resection that may enhance the ability to perform minimally invasive parenchymal-sparing operations.

## Graphical Abstract

**Figure:**
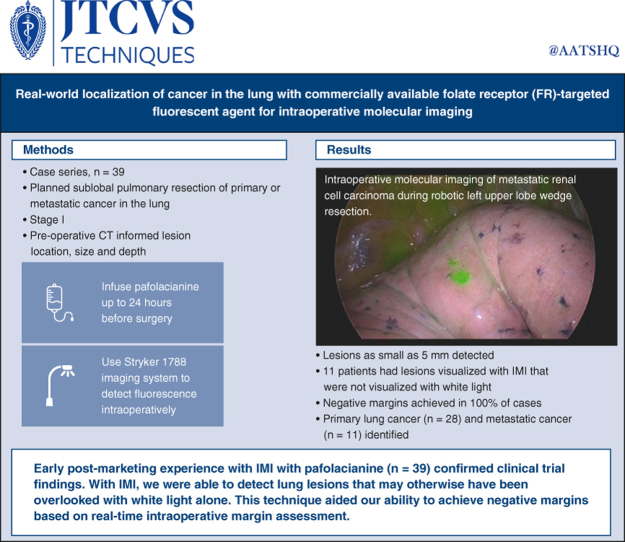

**Figure:**
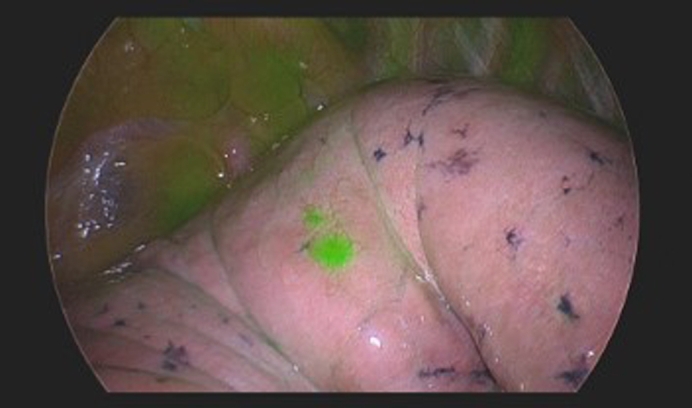
Lesion identification by IMI with pafolacianine in metastatic renal cell carcinoma.

**Central Message**

Real-world experience demonstrates the translatability and clinical utility of using intraoperative molecular imaging with pafolacianine for lesion visualization in lung cancer.

**Perspective**

With minimally invasive approaches, techniques to aid lung lesion identification may help surgeons attain negative margins when performing parenchymal-sparing procedures. Intraoperative molecular imaging with pafolacianine is an approved method for real-time lesion identification. This approach may facilitate identification of lesions and enhance performance of margin-negative, sublobar resections.

See Commentator Discussion on page 177.

Increasing use of minimally invasive surgical techniques, including video-assisted thoracoscopic surgery (VATS) and robotic-assisted thoracoscopic surgery (RATS), has the potential to decrease morbidity and improve postoperative recovery compared to open approaches.^1^, ^2^, ^3^, ^4^, ^5^ However, the increasing use of minimally invasive approaches may amplify challenges associated with surgical resection in non–small cell lung cancer.^6^ Surgical resection has a 15% to 20% local failure rate within the first 5 years after surgery among stage 1 patients, which often can be attributed to suboptimal detection, incomplete removal of all lesions, and/or failure to achieve negative margins using traditional approaches.^7^, ^8^, ^9^, ^10^, ^11^, ^12^, ^13^ Additionally, reductions in tactile feedback risks missing particularly small lesions and ground-glass opacities (GGOs).^6^

Reliable lung nodule identification, evaluation, and treatment planning are essential to the thoracic surgeon's ability to provide optimal patient outcomes. Traditional methods of percutaneous marking and, more recently, navigational bronchoscopy–guided dye marking support this important process yet are imperfect approaches.^14^, ^15^, ^16^, ^17^, ^18^, ^19^, ^20^ Either technique can aid localization when a lesion is readily accessible but are less reliable depending on lesion location. Moreover, they fail to provide any real-time intraoperative information for margin assessment. Novel approaches should be (1) readily adoptable by most medical centers, (2) intuitive to use for thoracic surgeons, and (3) not have an unreasonable need for additional specialty equipment and/or multidisciplinary coordination.^14^

Intraoperative molecular imaging (IMI) using pafolacianine may be an approach that meets these criteria. Specifically, this method includes intravenous preoperative administration of a tumor-specific receptor–targeted agent—pafolacianine, a novel fluorescent imaging agent that binds folate receptors—that can be visualized intraoperatively using near-infrared (NIR) imaging.^6^^,^^14^

Following December 2022 approval from the Food and Drug Administration for the use of pafolacianine for intraoperative molecular imaging of cancer in the lung, we sought to determine the translatability of clinical trial findings to the real world. We report on the outcomes of 39 consecutive cases of primary or metastatic disease of the lung at the University of Pittsburgh Medical Center.

## Materials and Methods

### Participants

Cases from June 2023 through January 2024 were reviewed. All patients who received pafolacianine during this time frame are included in this series (IRB STUDY20050006; approved July 23, 2020, with a waiver of consent).

Patients with primary lung cancer undergoing curative intent resection or resection of metastatic lesions of a nonpulmonary origin were included. A preoperative plan for sublobar resection was required. Lesion characteristics were determined based on preoperative computed tomography and positron emission tomography imaging. Patients were selected based on anticipated intraoperative challenges on nodule localization including small nodule size, deep location within the lung and subsolid makeup. There are no established cutoffs for lesion characteristics in the context of ability to visualize, so the potential use of the technology was left completely to the discretion of the surgeon and anticipated utility of IMI with pafolacianine.

The preoperative computed tomography scan closest to the date of surgery was used to determine lesion size, depth, and makeup. Lesion size was determined as the largest diameter on axial view. Lesion depth was defined as the shortest distance from a pleural edge to the border of the lesion. Lesion makeup (solid vs semisolid vs GGO) was determined by radiologic and/or surgeon interpretation.

### Drug Mechanism, Dosing, and Administration

Pafolacianine, a folate analog indocyanine green–like conjugate, is a fluorescent imaging agent that binds FR, internalizes via receptor-mediated endocytosis, accumulates intracellularly, and is eliminated from receptor-negative tissues with a half-life of <30 minutes. By accumulating preferentially in FR-positive tumors, pafolacianine can label nodules so they are visually highlighted intraoperatively when excited using an NIR lighting system. Pafolacianine absorbs light in the NIR region within a range of 760 nm to 785 nm, with peak absorption of 776 nm, and emits fluorescence within a range of 790 nm to 815 nm, with a peak emission of 796 nm.^6^

For each patient, 1 single-use vial of pafolacianine was thawed then shaken or vortexed for 60 seconds. Each vial contains 2 mg/mL concentrated drug solution; to achieve the target dose of 0.025 mg/kg, the body weight–based volume of concentrated drug was calculated for each patient, withdrawn from the vial, and added to 250 mL of 5% dextrose injection (USP bag). The infusion bag was then gently swirled by hand to mix for 1 minute and protected from light. Pafolacianine (0.025 mg/kg) was administered to the patient intravenously over a 60-minute infusion period within 24 hours of surgery.

### Surgical Methods and Fluorescent Imaging

The use of robotic or standard thoracoscopic modalities was left to surgeon discretion. Following entry into the chest, a standard white light camera system was used to inspect the pleura and lung surface. With lung manipulation, possible localization with white light was attempted. Subsequently, the lung was inspected with IMI using the Stryker 1788 imaging system, and a similar attempt was made at nodule localization. A standard hilar dissection was performed for anatomic resection. IMI was used during parenchymal division to assist with margin determination. Video 1 demonstrates intraoperative fluorescence detection and robotic methods. The margin was further assessed on the operating room back table with IMI of the specimen prior to sending to pathology. Frozen section analysis was performed on all main lesions resected. Frozen section analysis of occult lesions was left to the discretion of the surgeon. Patients were managed postoperatively through a standard lung resection pathway.

### Data Analyses

Patient demographic, operative, and final pathologic characteristics are reported. Continuous variables are reported as median and range; categorical variables, as frequency counts and percentages. There was no missing data and no imputation of data.

## Results

**Figure 1:**
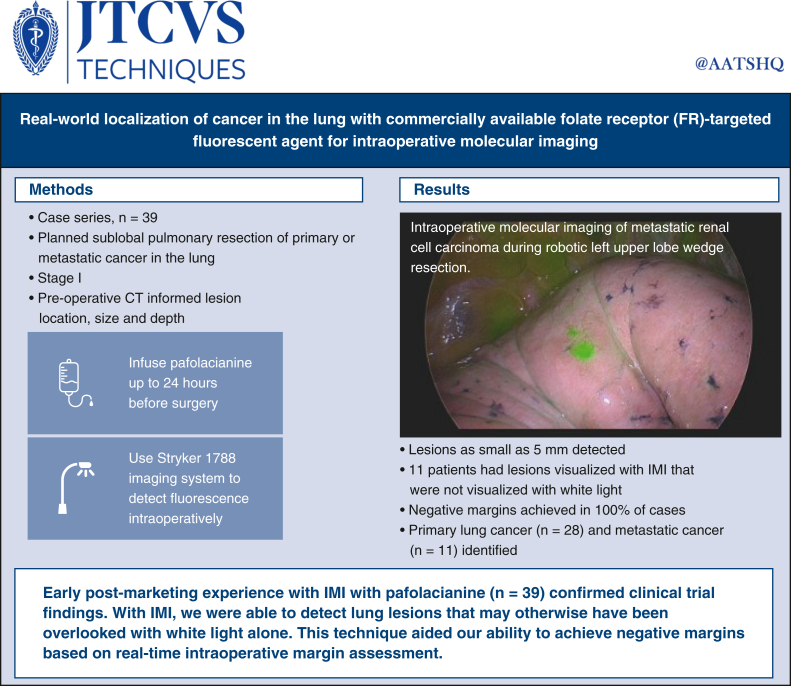
Visual summary of the methods, results, and implications of the reported case series. *FR*, Folate receptor; *IMI*, intraoperative molecular imaging.

A total of 39 patients met the study's inclusion criteria, with 28 females and 11 males. Their median age was 68 years (range, 41-87 years), and their median length of stay was 1.7 days. Figure 1 summarizes the methods and results of this case series.

### Primary Lung Cancer

Twenty-eight patients underwent resection of a primary lung cancer. Patient and operative characteristics are summarized in Table 1. The median size of all lesions was 13 mm (range, 6-32 mm). One-half of the lesions (14 of 28) were located in the right upper lobe. The primary lesion was successfully visualized with white light in 19 of the 28 patients (67.9%) and with IMI in 24 patients (85.7%). Eight patients (28.6%) had their lesions visualized with IMI but not white light. Attempts at palpation or indirect palpation were performed during the white light assessment before attempts at visualization with IMI. These same attempt at palpation or indirect palpation also was performed during the IMI assessment. A lesion identified with palpation or indirect palpation was recorded as visualized.

**Table 1:** Demographic and operative characteristics of the study cohort (N = 39)

Characteristic	Overall	Primary lung cancer	Metastatic
Patients, n	39	28	11
Age, y, median (range)	68 (41-87)	69 (44-87)	66 (41-79)
Sex, n (%)			
Female	28 (71.8)	22 (78.6)	6 (55.5)
Male	11 (28.2)	6 (21.4)	5 (45.5)
Length of stay, d, median (range)	1.7 (0.5-10.4)	2.4 (0.5-10.4)	1.5 (0.6-2.7)
Lesion location, n (%)			
Right upper lobe	16 (41.0)	14 (50.0)	2 (18.2)
Right middle lobe	1 (3.6)	1 (3.6)	0
Right lower lobe	6 (15.4)	4 (14.3)	2 (18.2)
Left upper lobe	11 (28.2)	5 (17.9)	6 (54.5)
Left lower lobe	5 (12.8)	4 (14.3)	1 (9.1)
Lesion size, mm, median (range)	13 (5-32)	13 (6-32)	13 (5-27)
Method of primary lesion visualization, n (%)			
White light only	3 (7.7)	3 (10.7)	0
IMI only	11 (28.2)	8 (28.5)	3 (27.2)
IMI and white light	24 (61.5)	16 (57.1)	8 (72.7)
Neither white light nor IMI	1 (2.6)	1 (3.6)	0
Surgical approach, n (%)			
Robotic	21 (53.8)	15 (53.6)	6 (54.5)
Thoracoscopic	18 (46.2)	13 (46.4)	5 (45.5)
Procedure performed, n (%)			
Segmentectomy	15 (38.5)	14 (50)	1 (9.1)
Wedge resection	17 (43.6)	8 (28.6)	9 (81.8)
Segmentectomy and wedge resection	3 (7.7)	2 (7.1)	1 (9.1)
Lobectomy	4 (10.3)	4 (14.3)	0
Pathology of primary lung cancer, n (%)			
Adenocarcinoma	N/A	23 (82.1)	N/A
Squamous cell carcinoma	N/A	2 (7.1)	N/A
Poorly differentiated carcinoma	N/A	1 (3.6)	N/A
Neuroendocrine tumor	N/A	2 (7.1)	N/A
Tissue origin of primary cancer, n (%)			
Lung	28 (71.8)	28 (100)	0
Colon	5 (12.8)	0	5 (45.5)
Breast	1 (2.6)	0	1 (9.1)
Cervical	1 (2.6)	0	1 (9.1)
Renal	1 (2.6)	0	1 (9.1)
Salivary duct	1 (2.6)	0	1 (9.1)
Urothelial	1 (2.6)	0	1 (9.1)
Necrotizing granuloma	1 (2.6)	0	1 (9.1)
Negative margin achieved in single surgery, n (%)	39 (100)	28 (100)	11 (100)

*IMI*, Intraoperative molecular imaging; *N/A*, not applicable.

Minimally invasive resection (RATS, n = 21; VATS, n = 18) was performed in all patients. Most patients underwent anatomic lung resection (segmentectomy, n = 15; wedge resection, n = 17; segmentectomy and wedge resection, n = 3). Patients had wedge resection performed for either multifocal ground-glass peripheral lesions or the intention of resecting metastatic lesions with an unexpected finding of primary lung cancer on final pathology. Four patients underwent lobectomy. In 2 patients, sublobar resection was planned but visualization with IMI revealed a likely inadequate margin, so lobectomy was performed. In 1 patient, a middle lobe lesion without a preoperative diagnosis was localized and removed via wedge resection. With intraoperative pathologic analysis revealing a primary lung cancer, completion lobectomy was performed, given the amount of additional parenchyma that had to be resected to achieve an adequate margin. In 1 patient, a segmentectomy was performed with an adequate margin, but intraoperative pathologic analysis of a hilar lymph node revealed metastatic disease.

All patients had a negative final margin, with 1 patient requiring an additional parenchymal resection margin. This close margin was visualized with ex vivo IMI on the operative back table, so an additional parenchymal resection was performed to obtain a better margin. The majority of tumors resected (n = 23; 82.1%) were adenocarcinomas.

### Metastatic Lesions

**Figure 2:**
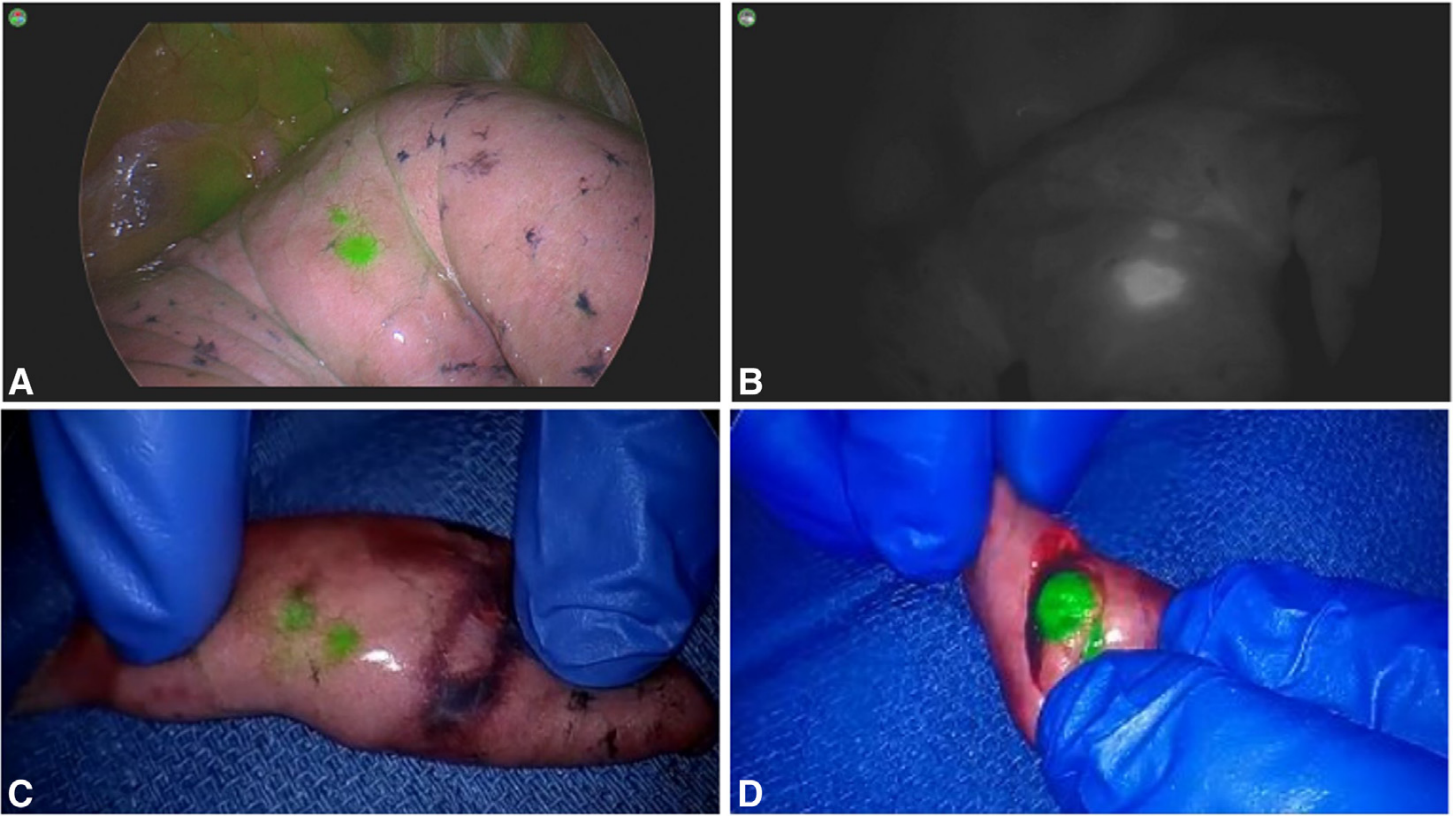
Case example: metastatic renal cell carcinoma. Robotic left upper lobe wedge resection of metastatic renal cell carcinoma using intraoperative molecular imaging (*IMI*) with pafolacianine. Lesion size, 5 mm. A and B, In vivo imaging with color overlay mode (A) and contrast mode (B). C and D, Ex vivo imaging.

Eleven patients underwent resection of metastatic lesions. Patient and operative characteristics are shown in Table 1. Six of the 11 lesions were in the left upper lobe (54.5%), with a median lesion size of 13 mm (range, 5-27 mm). Lesions were successfully identified with white light in 8 of the 11 patients (72.7%) and with IMI in 11 patients (100%). There were 3 patients (27.3%) with lesions visualized with IMI but not with white light. Figure 2 shows images of a lung lesion in a patient with metastatic renal cell carcinoma. An attempt at lesion palpation was made only in cases not visualized by white light or IMI.

Minimally invasive approaches were used in all 11 patients, with a robotic approach in 6 patients (54.5%) and a thoracoscopic approach in 5 patients (45.5%). Wedge resection was performed in most cases. A negative margin was achieved in all patients, with 1 patient requiring additional parenchymal resection. Final pathology demonstrated colon adenocarcinoma in 5 patients (45.4%). In 1 patient, metastatic thyroid cancer was suspected, with final pathology demonstrating necrotizing granuloma. This lesion was visualized with IMI.

### Occult Lesions

Seven patients (18%) had occult lesions identified, all of which removed via wedge resection. Malignant disease was found in 3 patients (primary lung adenocarcinoma, n = 2; metastatic cervical squamous cell carcinoma, n = 1). Atypical adenomatous hyperplasia was found in 2 patients, and lung parenchyma with inflammatory changes was detected in 2 patients.

### IMI Only

In 11 patients, the primary lesion was not detectable under visual inspection with white light or standard palpation techniques but was visualized with IMI only. The lesions detected by IMI only had an average size of 1.1 cm and an average depth of 1.2 cm. The lesions were classified as solid (n = 5), semisolid (n = 5), or GGO (n = 1).

## Discussion

This real-world experience demonstrates that intraoperative molecular imaging using pafolacianine allows for enhanced lesion identification, reliable intraoperative margin assessment, and identification of occult lesions all while using minimally invasive surgical approaches. Specifically, we observed 28.2% of lesions localized with this novel approach that were not identified with standard white light imaging. All patients in this case series had negative final margins. Occult lesions were resected from nearly 18% of the patients. These findings are consistent with those reported from the phase 3 ELUCIDATE clinical trial, in which 29.2% of patients had a primary lesion identified with IMI that was not found with white light only, and occult synchronous lesions were found in 8% of study participants.^6^ Implementation of this approach into clinical practice was shown to be successful in this case series, thereby demonstrating the translatability of this approach outside of a controlled trial setting. Most importantly, these observations allow us to consider practical applications of intraoperative molecular imaging in a routine clinical setting of care.

Successful identification of any nodule in the lung, whether primary cancer or metastatic disease, is of central importance. IMI has some important unique characteristics compared to traditional methods of robotic bronchoscopic or image-guided dye marking and wire localization. Robotic bronchoscopy with dye marking must be performed in the operating room before resection, thereby adding to the overall operative time for each case. During dye marking, many nodules are not visualized directly, running the risk of missing a nodule and applying dye to an incorrect location or the pleural space, impairing the desired precision of nodule localization. In contrast, IMI with pafolacianine uses a targeted agent administered outside of the operating room via intravenous injection. The overall result is decreased operating time and complexity without reliance on additional procedures, as well as more targeted nodule localization compared to existing methods. Furthermore, the intravenous administration of pafolacianine enables simultaneous labeling of multiple lesions of various sizes, avoiding the need to prioritize a single lesion labeled with dye.

There are several possibilities to consider when exploring the 14% of primary lung cancer cases in which the lesion was not visualized with IMI. The ability of IMI with pafolacianine to fluoresce a lesion often is multifactorial and patient-dependent. Such variables as patient history, inflammatory processes, imaging system used, previous resections, size, depth, anatomic location, port placement, and other factors need to be considered. Moreover, goals of the procedure should be identified, to assess the use of pafolacianine as an adjunct to aid localization, identification of occult disease, or margin assessment. Successful visualization relies on the interplay between drug administration and use of specific camera technology. Achieving the optimal camera position based on lesion depth and location may require changing angles, or port sites. Interestingly, in most cases in which the lesion was not visualized in vivo, it could be visualized with IMI within the specimen ex vivo on the operative back table. This further supports the idea that unsuccessful lesion visualization is more likely associated with the need for technical optimization and not with a lack of drug uptake within the lesion.

Additionally, consideration can be given to lesions that might not express the folate receptor required for drug binding. Approximately 85% of lung and pleural malignancies contain FR-positive nodules, thus making FRs ideal targets for imaging agents such as pafolacianine. The list of histologies found to express FR-α, FR-β, or both is expanding. The phase 3 trial results demonstrated a range of histologies from both primary and pulmonary metastases including adenocarcinoma, squamous cells, adenosquamous cells, and others.^6^ The use of folate-containing supplements may reduce the binding of pafolacianine to FRs, which could reduce lesion detection. In general, it is recommended that patients avoid folate-containing supplements within 48 hours of pafolacianine administration.

Historically, the successful localization of small (subcentimeter) lesions relied on palpation achieved through open approaches, such as the Perelman technique.^21^^,^^22^ In many ways, this level of feedback was sacrificed with VATS approaches and even more so with RATS, in which there is no level of tactile feedback. IMI with pafolacianine is a minimally invasive method that preserves the ability to detect lesions of various sizes. As shown from our experience, we detected lung lesions as small as 5 mm using this approach.

Demonstrating the successful use of IMI to detect especially small, subsolid lesions raises an important consideration. In the case of multifocal GGOs, we typically identify and resect only the most suspicious lesion and leave the rest to be monitored over time, because many would be difficult to localize. Pure GGOs can be detected with this technology and has been an important part of our experience and the intraoperative utility of this product. With IMI, the enhanced ability to identify lesions should not necessarily translate to resection of more lesions. While IMI provides greater visualization and additional information about the lesions, the surgeon must take into consideration the patient's clinical history, possibility of a false-positive finding, and implications of resection to inform the ultimate surgical plan.

In addition to accurate lesion identification, it also is important to successfully achieve negative margins. All patients in our case series had negative final margins, with only 2 of 39 requiring additional parenchymal resection, performed during the same operation as a result of real-time intraoperative assessment. Sublobar resection has been shown to be noninferior to lobectomy for non–small cell lung cancer, and thus increasing interest in and implementation of sublobar procedures for early-stage cancers can be expected.^23^^,^^24^ A challenging aspect of this trend is that sublobar resection is a more technically demanding approach, with surgeons needing to reliably identify the intersegmental plane while achieving adequate margins.^25^ The use of IMI can help inform surgeons of sufficient distances from tumors and even can aid intraoperative decision making regarding the feasibility of performing sublobar resection.

This study has several limitations. While some data were collected prospectively, this was a retrospective single-center study with a limited sample size. As experience with this technology grows, opportunities for multicenter collaborative studies will help elucidate the optimal clinical applications. Additionally, it is known that the systemically administered drug can be visualized in the lymphatics due to activated macrophages, which express FR-β. For this reason, a lymph node may fluoresce in the absence of cancer, so clinical use of pafolacianine should be limited to lung nodules.

As we continue to gain experience with IMI in lung cancer patients, we have identified several practical applications of IMI in clinical practice. One use is for the identification of small peripheral nodules needing diagnosis and the identification of occult disease. Others include the localization of GGOs and guidance for appropriate resection, localization for wedge resections and segmentectomy to provide real time in vivo margin assessment to guide stapling, and identification of and clinical decision making related to lesions on the border zone of segmental planes or that cross a fissure.

## Conclusions

This case series demonstrates the translatability of the novel method of IMI with pafolacianine from clinical research to clinical practice. Furthermore, these data confirm the findings from clinical trials, showing the ability of IMI to identify lesions not visible by white light alone and its utility for real-time margin assessment during surgery. Incorporation of IMI into clinical practice has the potential to improve outcomes for patients with lung cancer.

### Webcast

You can watch a Webcast of this AATS meeting presentation by going to: https://www.aats.org/resources/first-reported-real-world-use-7339.

**Figure:**
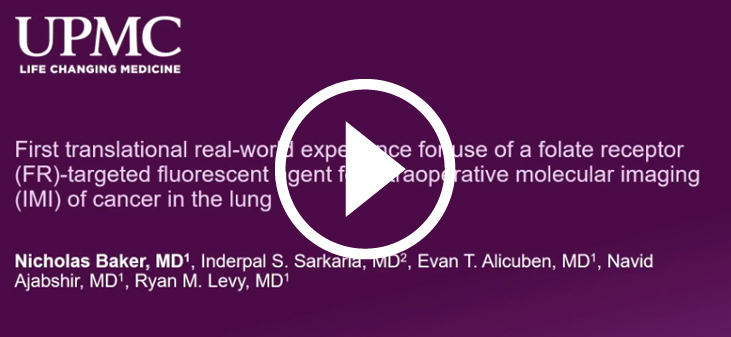

## Conflict of Interest Statement

Dr Baker is a speaker for Stryker Medical. All other authors reported no conflicts of interest.

The *Journal* policy requires editors and reviewers to disclose conflicts of interest and to decline handling or reviewing manuscripts for which they may have a conflict of interest. The editors and reviewers of this article have no conflicts of interest.

## Supplementary Data

Video 1 Demonstration of lung parenchyma with intraoperative molecular imaging (IMI) and without IMI. The borders of the visualized nodule are used to guide stapled margin creation. Video available at: https://www.jtcvs.org/article/S2666-2507(25)00039-2/fulltext.
