# Supplementary figures and images for: Real-world localization of cancer in lungs with a commercially available folate receptor-targeted fluorescent agent for intraoperative molecular imaging
Source: JTCVS Tech. 2025 Jan 23;31:161–8. doi: 10.1016/j.xjtc.2024.12.011 (PMC12237771; doi:10.1016/j.xjtc.2024.12.011)

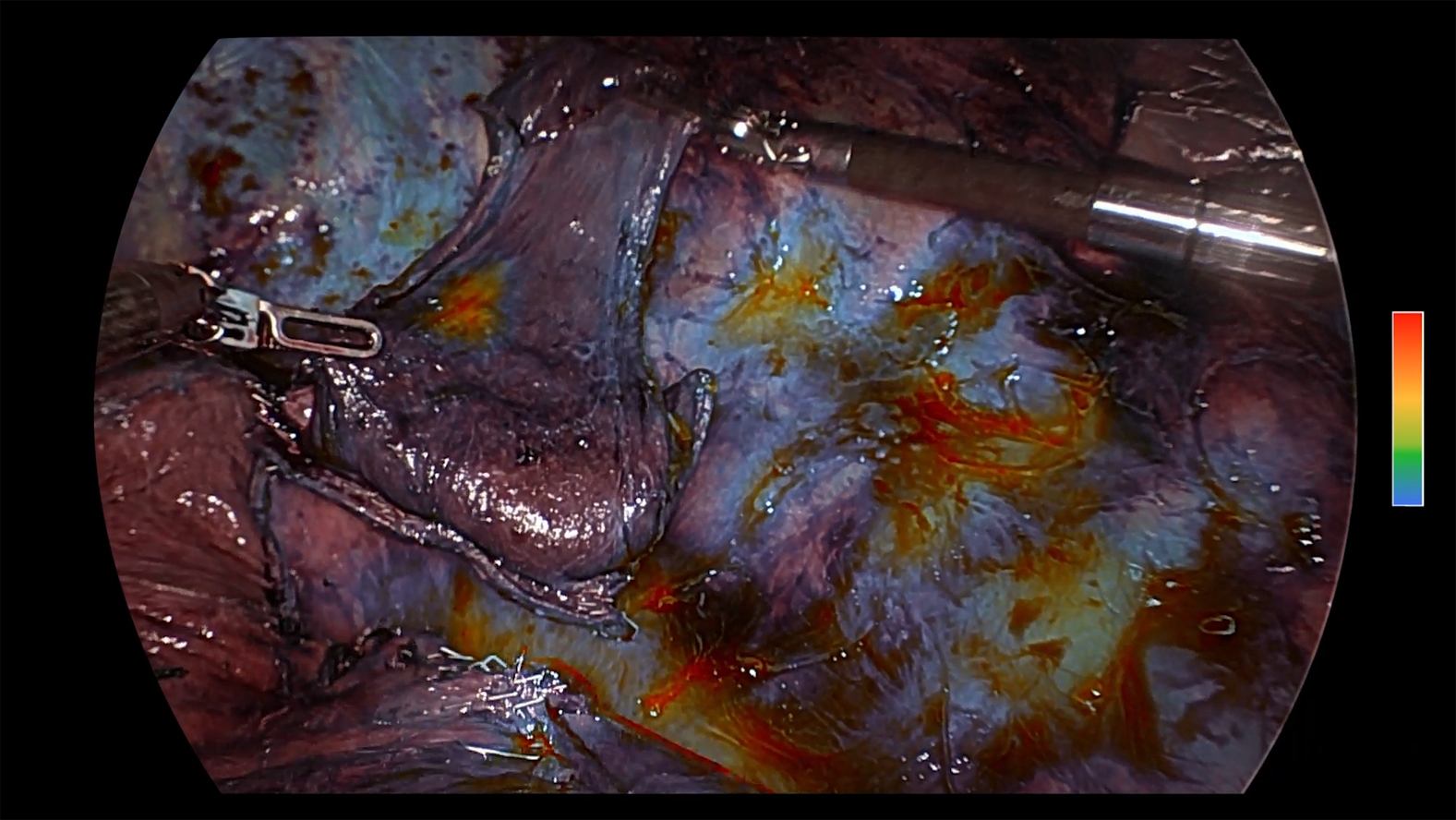

Supplement: Video 1 — Demonstration of lung parenchyma with intraoperative molecular imaging (IMI) and without IMI. The borders of the visualized nodule are used to guide stapled margin creation. Video available at: https://www.jtcvs.org/article/S2666-2507(25)00039-2/fulltext. [file fx3.jpg]
